# Aptamer Combined with Fluorescent Silica Nanoparticles for Detection of Hepatoma Cells

**DOI:** 10.1186/s11671-017-1890-6

**Published:** 2017-02-07

**Authors:** Zixi Hu, Juntao Tan, Zongqiang Lai, Rong Zheng, Jianhong Zhong, Yiwei Wang, Xiaoxue Li, Nuo Yang, Jieping Li, Wei Yang, Yong Huang, Yongxiang Zhao, Xiaoling Lu

**Affiliations:** 1 National Center for International Research of Biological Targeting Diagnosis and Therapy, Guangxi Key Laboratory of Biological Targeting Diagnosis and Therapy Research, Collaborative Innovation Center for Targeting Tumor Diagnosis and Therapy, Guangxi Medical University, Nanning, Guangxi China; 2 Surgery Oncology Department, Affiliated Tumor Hospital of Guangxi Medical University, Nanning, China; 3 The Department of Immunology, Guangxi Medical University, Nanning, Guangxi China

**Keywords:** Aptamer, Fluorescent nanoparticles, Hepatoma, Cancer

## Abstract

**Purpose:**

The purpose of this study is to develop a simple, effective method to label hepatoma cells with aptamers and then detect them using fluorescent silica nanoparticles (FSNPs).

**Method:**

Streptavidin was conjugated to carboxyl-modified fluorescein isothiocyanate (FITC)-doped silica nanoparticles which were prepared by the reverse microemulsion method. The resulting streptavidin-conjugated fluorescent silica nanoparticles (SA-FSNPs) were mixed with hepatoma cells that had been labeled with biotin-conjugated aptamer TLS11a (Bio-TLS11a). The specificity and sensitivity of the nanoprobes were assessed using flow cytometry and fluorescence microscopy. Their toxicity was assessed in normal human liver cell cultures using the MTT assay, as well as in nude mice using immunohistochemistry.

**Results:**

SA-FSNPs showed uniform size and shape, and fluorescence properties of them was similar to the free FITC dye. SA-FSNPs were able to detect aptamer-labeled hepatoma cells with excellent specificity and good sensitivity, and they emitted strong, photobleach-resistant fluorescent signal. SA-FSNPs showed no significant toxic effects in vitro or in vivo.

**Conclusion:**

The combination of biotin-conjugated aptamers and SA-FSNPs shows promise for sensitive detection of hepatoma cells, and potentially of other tumor cell types as well.

## Background

Early diagnosis of cancer is key to improving the survival and prognosis of cancer patients [1]. Most cancer detection methods, including blood biochemistry, genetic analysis, and imaging have disadvantages such as low sensitivity, high false-positive rates, high cost or complex procedures [2, 3]. Thus, researchers continue to investigate ways to detect tumor cells simply and effectively in early stages of cancer.

While traditional antibodies against tumor markers can aid in cancer diagnosis, recently developed “chemical antibodies”, which are short sequences of single-stranded DNA or RNA known as aptamers, may prove to be superior. Aptamers specifically recognize targets such as small molecules, protein, virus, bacteria, and whole cells [4, 5]. Aptamers can show higher selectivity and affinity, as well as lower immunogenicity, than traditional antibodies; aptamers are also easier to synthesize, and they can penetrate tissue more rapidly with fewer toxic effects [5–7]. Hundreds of aptamers against tumor cells, most of them labeled with organic dyes, have been described for tumor cell detection [8–13]. One disadvantage of using these fluorescent dye labeled aptamers on their own is that they are rapidly photobleached, severely hindering their clinical usefulness [14].

Recently, the functionalized silica nanoparticles for biosensing have attracted the interest of many researchers [15–18]. And one way to reduce photobleaching of fluorescent-dye labeled aptamers is to conjugate aptamers to the surface of fluorescent silica nanoparticles (FSNPs) [19–21]. With their unique core-shell structure, FSNPs show good biocompatibility, chemical stability, and photostability [22]. Many aptamer-functionalized FSNPs have been reported that they detect tumor cells and show clinical potential for cancer diagnosis [23–25]. However, linking aptamers directly to the nanoparticle surface may destabilize the nanoparticles by making them so large that they are cleared from the circulation [26]. It may also limit the specificity and selectivity of aptamer targeting because of steric hindrance between the target tumor cells and the nanoparticles, such as when aptamer DNA “lies down” on the nanoparticle surface [27]. This is indeed the case with anti-tumor antibodies, which lose much of their sensitivity and specificity after being conjugated to nanoparticles [28].

**Fig. 1:**
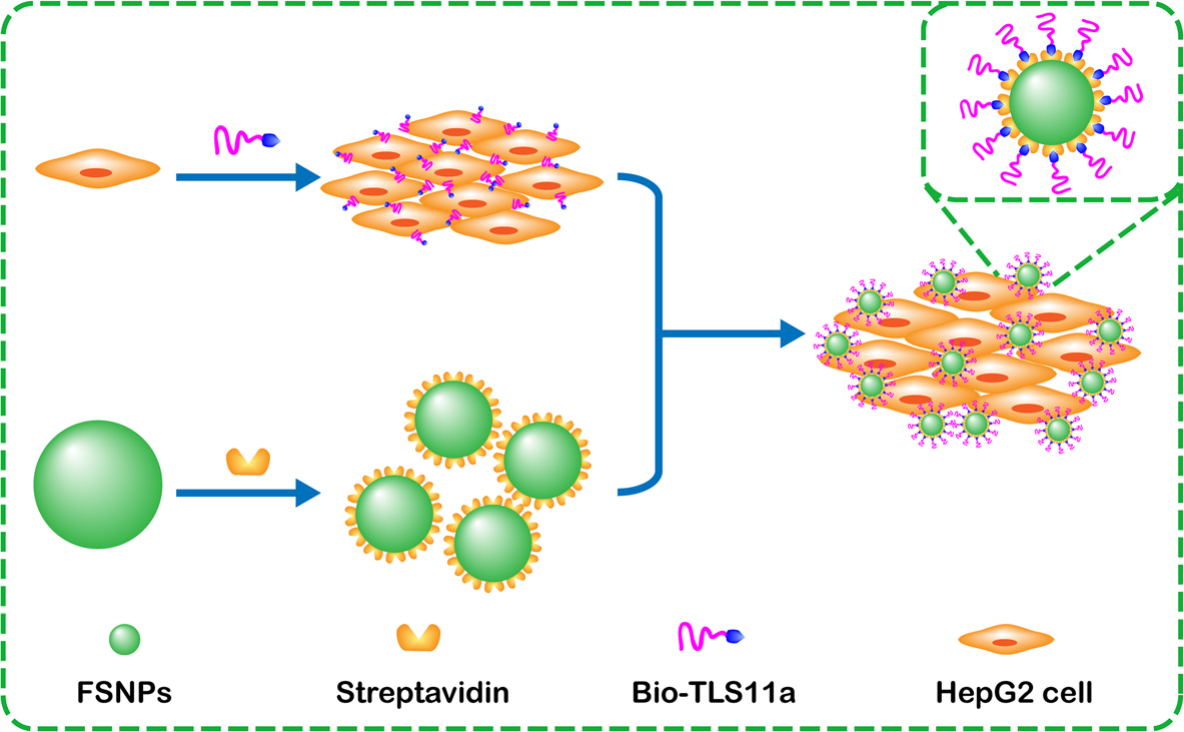
Schematic illustration of highly sensitive detection of HepG2 hepatoma cells using a biotin-conjugated aptamer (Bio-TLS11a) and streptadivin-conjugated fluorescent silica nanoparticles (FSNPs)

To avoid these potential problems arising from conjugating aptamers directly to FSNPs, we have developed an alternative approach in which the aptamer and FSNP are physically separate but interact via extremely strong biotin-streptavidin interaction (Fig. 1). HepG2 cells are incubated first with biotin-labeled TLS11a aptamer (Bio-TLS11a) and then with streptavidin-conjugated FSNPs (SA-FSNPs). The SA-FSNPs then bind and interact with cells where the biotin-labeled aptamer has bound. This approach avoids the limitations intrinsic to nanoparticle surface modification, and it may allow efficient, sensitive detection of cancer cells in vitro.

## Methods

### Cells and Animals

Human hepatoma cell line HepG2, human normal liver cell line L02, and human embryonic kidney cell line 293T were purchased from the Cell Bank of the Chinese Academy of Sciences (Shanghai, China). All cell lines were cultured at 37 °C under 5% CO_2_ in DMEM supplemented with 10% fetal bovine serum (FBS, Hyclone) and penicillin-streptomycin (Gibco, Grand Island, NY, USA).

Female BALB/c nude mice aged 4–6 weeks were obtained from the Guangxi Laboratory Animal Center (Guangxi, China) and housed in laminar flow cabinets under pathogen-free conditions. All experimental protocols were approved by the Animal Ethics Committee of Guangxi Medical University (Nanning, Guangxi, China).

### Reagents

Fluorescein isothiocyanate (FITC), cyclohexane, Triton X-100, *n*-hexanol, bovine serum albumin (BSA), acetone, tetraethyl orthosilicate (TEOS), (3-aminopropyl) triethoxysilane (APTES), 3-aminopropylmethyldimethoxysilane (APTMS), 1-ethyl-3-(3-dimethylaminopropyl) carbodiimide hydrochloride (EDC) N-hydroxysulfosuccinimide sodium salt (sulfo-NHS) and polyoxymethylene were bought from Sigma (St. Louis, MO, USA). Ethanol, dimethyl sulfoxide (DMSO), 3-(4,5-dimethylthiazol-2-yl)-2,5-diphenyltetrazolium bromide (MTT), and hematoxylin-eosin (HE) were purchased from Solarbio (Beijing, China). The nuclear dye 4′,6-diamidino-2-phenylindole (DAPI) was purchased from Life Technologies (USA). The biotin-labeled aptamer 5′-bio-(CH2)6-AGTAATGCCCGGTAGTTATTCAAAGATGAGTAGGAAAAGA-3′ (Bio-TLS11a) and FITC-labeled aptamer 5′-FITC-AGTAATGCCCGGTAGTTATTCAAAGATGAGTAGGAAAAGA-3′ (FITC-TLS11a) were synthesized by Shanghai Sangon Biotechnology (Shanghai, China).

### Preparation and Characterization of SA-FSNPs

FITC-doped, carboxyl-modified FSNPs were synthesized as described [14, 29, 30]. Briefly, a water-in-oil microemulsion was prepared with FITC, cyclohexane, Triton X-100, *n*-hexanol, and distilled water, giving rise to FITC-doped silica nanoparticles. These FSNPs were amine-modified using TEOS and APTES; the flocculent precipitate was collected by centrifugation and washed with acetone, followed by deionized water. The precipitate (2 mg) was dissolved in 1 mL of 0.1-M phosphate-buffered saline (PBS, pH 7.4) containing EDC (1 mg) and sulfo-NHS (2.5 mg). When the reaction was complete, 50 μl of streptavidin diluted in PBS was added to the solution, which was incubated at room temperature for 4 h with gentle shaking. The nanoparticles were washed with PBS and then resuspended in 1 ml of 0.05% BSA for 30 min to block free carboxylates, generating SA-FSNPs. The SA-FSNPs were washed three times with PBS and stored at 4 °C. For subsequent experiments, the SA-FSNPs were resuspended in PBS as needed.

The morphology and size distribution of SA-FSNPs were assessed using transmission electron microscopy (TEM; H-7650, Japan). Their photoluminescence was measured using a fluorescence spectrophotometer (FL-7000, Perkin Elmer, USA).

### Flow Cytometry of Aptamer-Labeled Cells Mixed with SA-FSNPs

HepG2 or L02 cells (3.0 × 10^5^ cells/ml) were harvested, washed three times with PBS, then incubated with for 30 min either with SA-FSNPs (ca. 0.1 mg, 1 ml) at room temperature or with FITC-TLS11a (100 nM) on ice. In either case, the cells and labeling agents were suspended in binding buffer (200 μl) prepared by supplementing PBS with 4.5 g/L of glucose and 5 mM of MgCl_2_. Other cell suspensions were incubated with Bio-TLS11a (100 nM) at 4 °C for 30 min, followed by SA-FSNPs (ca. 0.1 mg, 1 ml) at 37 °C for 60 min with gentle shaking. All suspensions were washed three times with PBS, suspended in 500 μl of binding buffer, and then analyzed by flow cytometry (Epics XL, Beckman Coulter, USA) using FLOWJO 7.6 software.

### Fluorescence Microscopy of Aptamer-Labeled Cells Mixed with SA-FSNPs

HepG2 and L02 cells were cultured for 12 h in 6-well plates (3 × 10^5^ cells per well). Cells were washed three times with cold PBS, fixed for 15 min with 4% polyoxymethylene, washed with PBS, and then incubated with SA-FSNPs or FITC-TLS11a, or the sequential combination of Bio-TLS11a followed by SA-FSNPs as described above. Finally, cells were stained with DAPI for 90 s, washed with PBS, and analyzed by fluorescence microscopy (DS-Ri1; Nikon Corporation, Tokyo, Japan). Fluorescence intensity was quantitated using Image Pro (Media Cybernetics, Bethesda, MD, USA).

### In Vitro Toxicity of SA-FSNPs

Toxicity of SA-FSNPs against 293T or L02 cells was assessed using the MTT assay. Cells (2 × 10^5^ /ml) were cultured overnight in 96-well plates, then treated with SA-FSNPs (0.1, 0.5, or 1.0 mg/ml) for 12, 24, or 48 h. Control cells were treated with PBS. At specific time points, 10 μl of MTT (5 mg/ml) was added to wells, and plates were incubated at room temperature for 4 h in the dark. The medium was discarded, 150 μl of DMSO was added to each well, and plates were incubated for 10 min. Optical density (OD) at 570 nm was measured using an ELISA microplate reader (Thermo Scientific, USA). Cell viability was calculated using the formula:

$$ \mathrm{Viability}\ \left(\%\right) = \mathrm{O}{\mathrm{D}}_{\mathrm{experimental}}/\mathrm{O}{\mathrm{D}}_{\mathrm{control}} \times 100\ \%. $$

### In Vivo Toxicity of SA-FSNPs

Nude mice received a single tail vein injection of 200-μl PBS or SA-FSNPs (1 mg/ml) (*n* = 3 animals per group). After 1 week, the animals were sacrificed, and the major tissues (heart, lung, liver, spleen, kidney) were immersed in 10% formaldehyde solution, dehydrated, and paraffin-embedded. Paraffin sections (4 μm thick) were processed using routine methods and stained with HE.

### Statistical Analyses

Statistical analysis was performed using Student’s *t* test and analysis of variance (ANOVA) in GraphPad Prism software (San Diego, CA, USA), with *P* < 0.05 defined as the significance threshold. Data were shown as mean ± SD or as median (range).

## Results and Discussion

Here, we explored the possibility of detecting human hepatoma HepG2 cells, a common cell model for liver cancer studies, using aptamer TLS11a, which was originally selected through the SELEX method to bind specifically to HepG2 cells and which shows promise for targeted diagnostics and therapy of hepatocellular carcinoma [10, 31–33]. In contrast to previous approaches in which the aptamer was conjugated to the surface of FSNPs, potentially limiting the sensitivity of aptamer-based detection, we kept the aptamer and FSNPs physically separate but we conjugated the former to biotin and the latter to streptavidin to allow for strong, specific interaction. Separating aptamer binding to target cells from FSNP binding to aptamer may allow a larger number of aptamers to bind to each target cell, amplifying the fluorescence signal.

### Characterization of SA-FSNPs

**Fig. 2:**
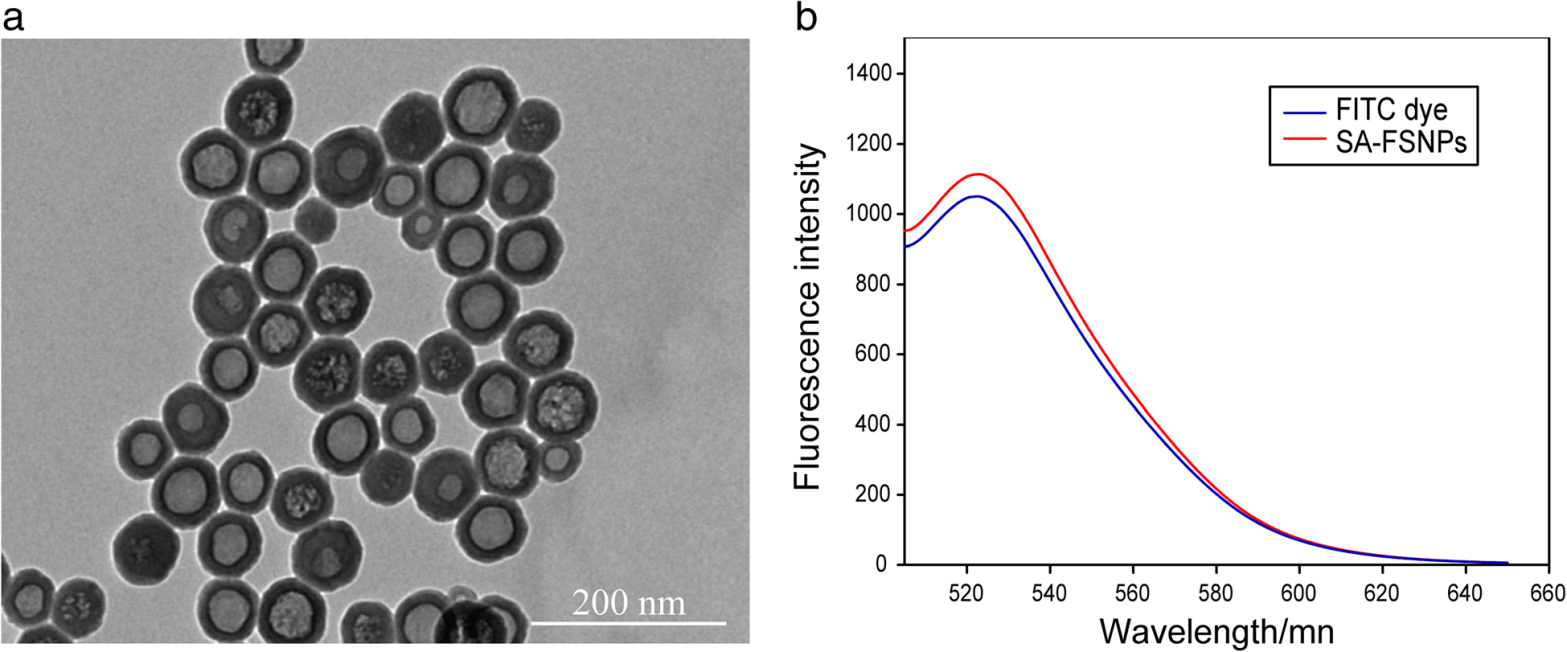
Characterization of SA-FSNPs. **a** Transmission electron micrograph of SA-FSNPs. **b** Fluorescence emission spectra of FITC dye and SA-FSNPs

Transmission electron microscopy showed SA-FSNPs to be nearly monodisperse and spherical, with an average diameter of 75.47 ± 2.52 nm (Fig. 2a). The core-shell structure of silica nanoparticles allows fluorescent dyes such as FITC to be trapped inside [34, 35]. Using rhodamine B in ethanol solution as a reference [36], the fluorescence quantum yields of FITC dye-doped silica nanoparticles were about 0.52. The maximum emission wavelength of free FITC dye and SA-FSNPs was 522 and 525 nm, respectively (Fig. 2b). The emission peak of SA-FSNPs is slightly red-shifted from FITC, which may be due to the loss of energy due to the interaction of silica substrate with the dye [37].

### Flow Cytometry of aptamer-Labeled Cells Mixed with SA-FSNPs

**Fig. 3:**
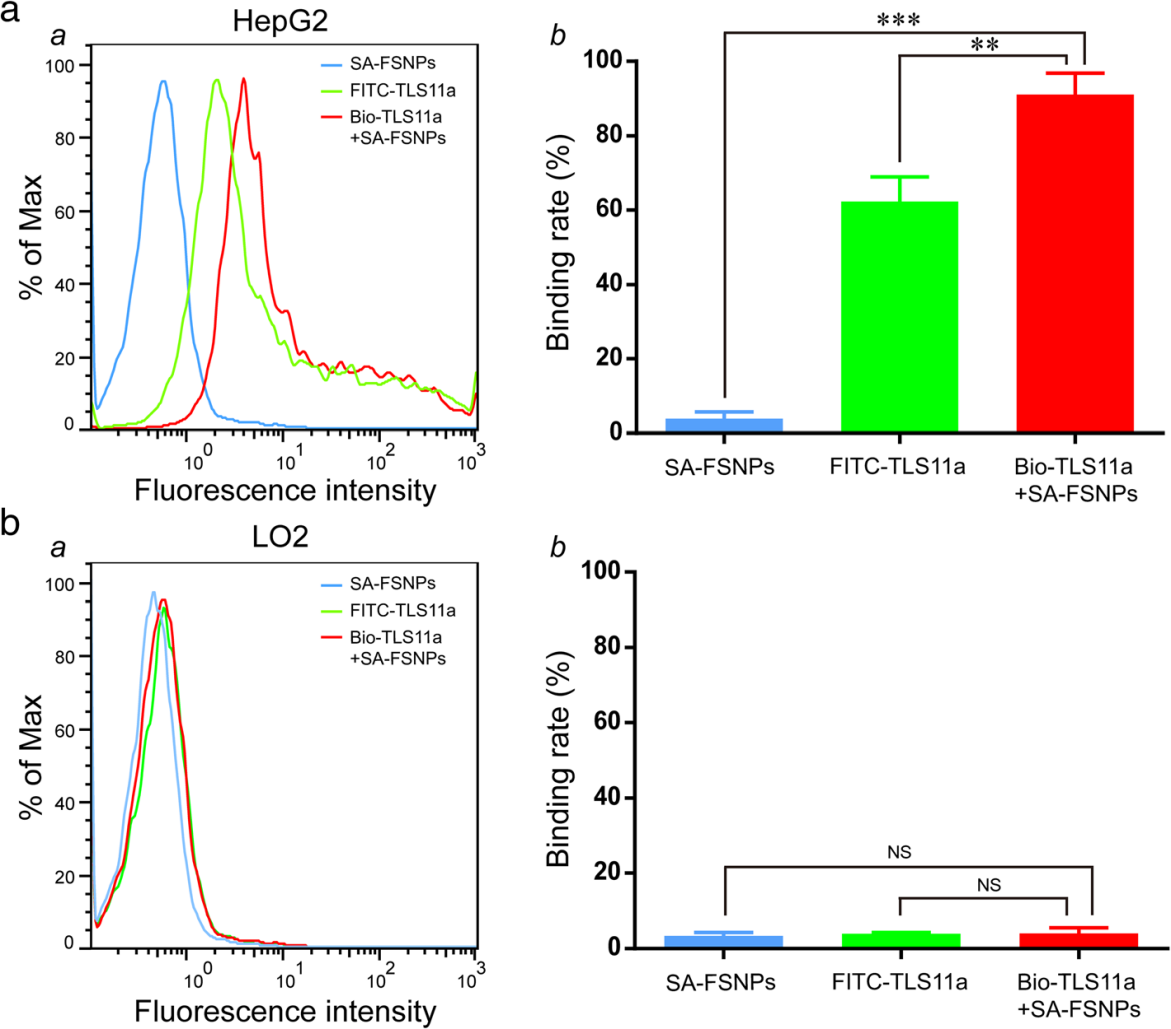
**a** Flow cytometric detection of HepG2 cells (*a*) or L02 cells (*b*) after incubation with SA-FSNPs or FITC-TLS11a or the combination of Bio-TLS11a and SA-FSNPs. **b** Quantitative analysis of HepG2 cells (*a*) or L02 cells (*b*). *NS* not significant. ^**^ *P* < 0.01, ^***^ *P* < 0.001

To determine whether the synthesized SA-FSNPs can be used as a detection probe for aptamer-labeled cells, HepG2 cells were firstly reacted with Bio-TLS11a. After washing, HepG2 cells was then incubated with SA-FSNPs. L02 cells served as negative cells, and FITC-TLS11a was used as a control probe. For the detection of HepG2 cells, stronger fluorescence intensity was found on Bio-TLS11a combined with SA-FSNPs (Bio-TLS11a + SA-FSNPs) and FITC-TLS11a, while no obvious fluorescence signal was observed on SA-FSNPs alone (Fig. 3a, panel *a)*. Statistical graph of the binding rate of HepG2 cells showed the similar results (Fig. 3a, panel *b)*. Additionally, there was no fluorescence signal on L02 cells after treating with SA-FSNPs alone, FITC-TLS11a and Bio-TLS11a + SA-FSNPs, respectively (Fig. 3b, panel *a*), in accordance with the results of statistical graph of the binding rate of L02 cells (Fig. 3b, panel *b*). These results suggest that the sequential combination of Bio-TLS11a with SA-FSNPs can detect HepG2 cells with higher specificity than FITC-TLS11a.

### Fluorescence Microscopy of Aptamer-Labeled Cells Mixed with SA-FSNPs

**Fig. 4:**
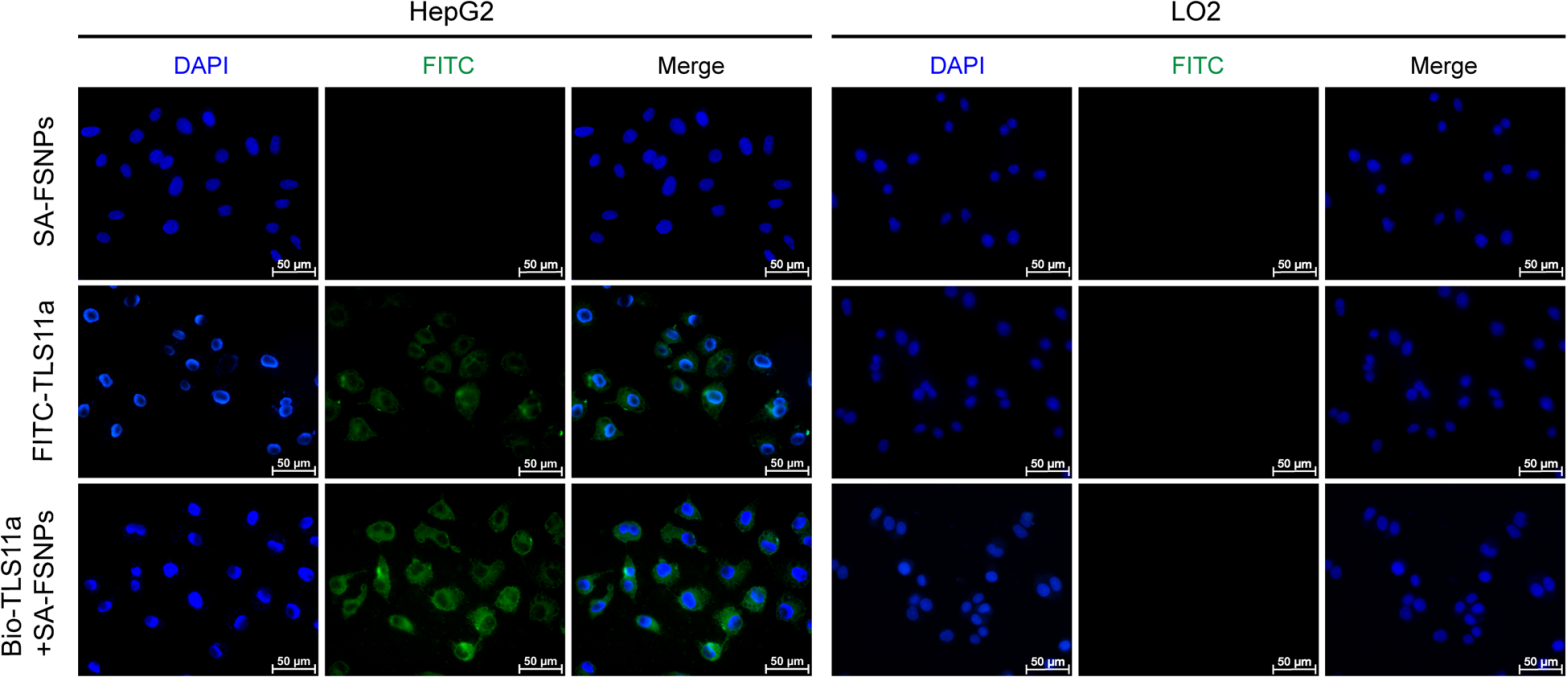
Fluorescence micrographs of HepG2 and L02 cells after incubation with SA-FSNPs or FITC-TLS11a or the combination of Bio-TLS11a and SA-FSNPs. SA-FSNPs and FITC were examined in the *green channel*, while DAPI-stained nuclei were examined in the *blue channel*

To allow a more direct visualization of HepG2 detection using our system, we used fluorescence microscopy to examine HepG2 cells incubated with SA-FSNPs or FITC-TLS11a or Bio-TLS11a + SA-FSNPs. As can be seen distinctly in fluorescence images, both FITC-TLS11a and Bio-TLS11a + SA-FSNPs showed green fluorescence on periphery of HepG2 cells, while SA-FSNPs did not. Furthermore, the fluorescence intensity of Bio-TLS11a + SA-FSNPs was stronger than FITC-TLS11a (Fig. 4a). No green fluorescence was observed on L02 cells after incubation with SA-FSNPs alone, FITC-TLS11a and Bio-TLS11a + SA-FSNPs, respectively (Fig. 4b), which was consistent with the analysis of flow cytometry. Therefore, we could agree that aptamer TLS11a could recognize and bind HepG2 cells with high affinity and specificity. Moreover, fluorescence signal from HepG2 cells is owing to the interaction between Bio-TLS11a labeled HepG2 cells and the SA-FSNPs. The SA-FSNPs display stronger fluorescent signals than the FITC-labeled aptamer probably due to the special core-shell structure of silica nanoparticles which allow the fluorescent dyes entrapped inside to prevent them from photodamaging oxidation [38–42].

### Photostability of SA-FSNPs

**Fig. 5:**
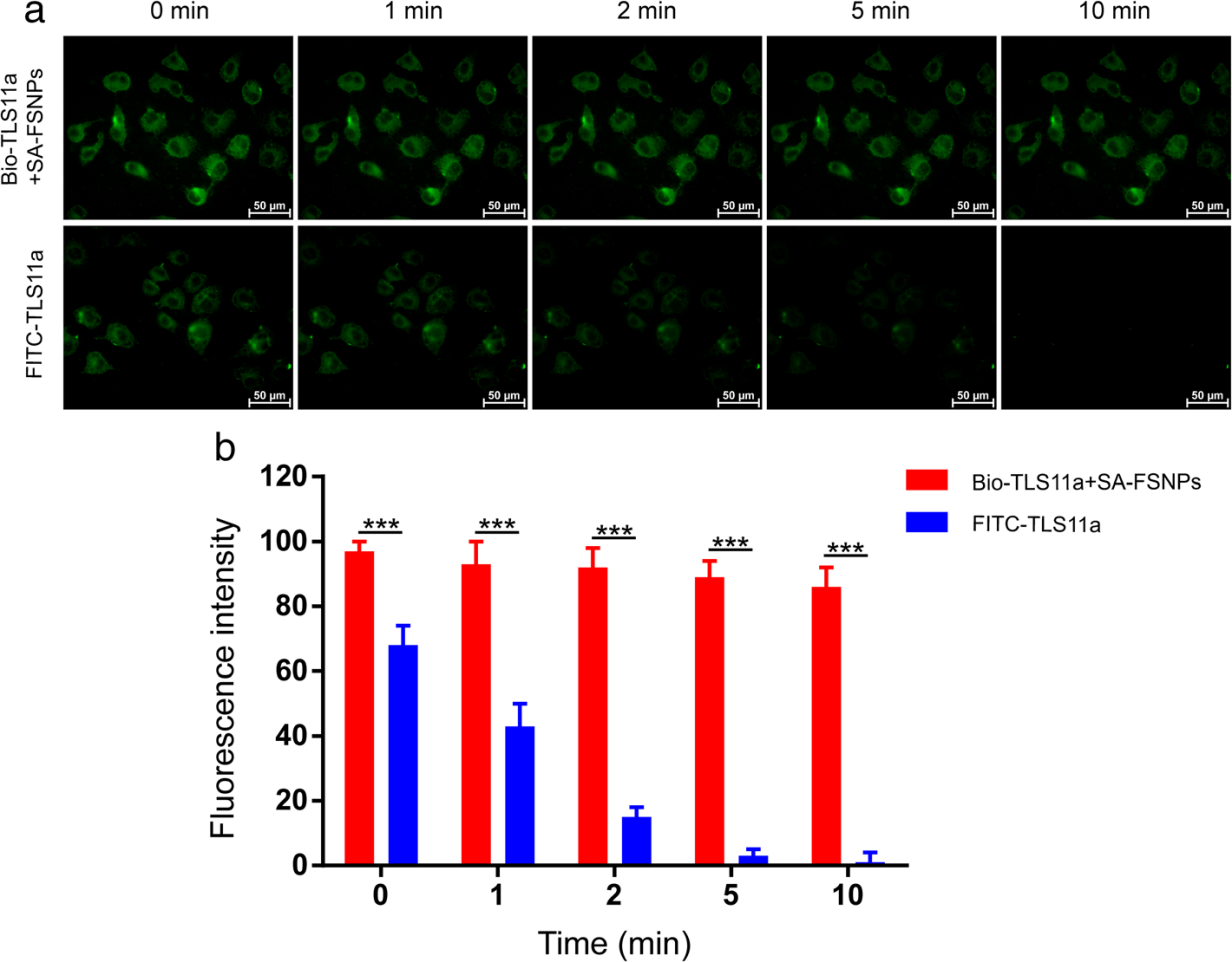
Photostability of FITC-TLS11a and of the combination of Bio-TLS11a with SA-FSNPs. **a** Fluorescence micrographs of HepG2 cells labeled with the combination of Bio-TLS11a and SA-FSNPs (*upper row*) or with FITC-TLS11a alone (*lower row*) and then continuously irradiated for the indicated periods. **b** Quantitative analysis of fluorescence intensity after different irradiation periods. ^***^ *P* < 0.001

Fluorescent dye molecules can quench easily after irradiation, limiting their usefulness. Doping fluorophores within porous silica nanoparticles can improve their photostability while maintaining their strong fluorescence emission [34, 35]. We measured the photostability of SA-FSNPs by mixing them with aptamer-labeled HepG2 cells and imaging the cells by fluorescence microscopy after continuous illumination lasting 0, 1, 5, and 10 min. In parallel, cells treated with FITC-aptamer alone were imaged in the same way. Green fluorescence from SA-FSNPs remained clearly visible even after intense irradiation for 10 min, whereas fluorescence from FITC-TLS11a had nearly disappeared after 2 min (Fig. 5). These results are consistent with the idea that fluorescent dye molecules are encapsulated within the silica matrix, where they are kept separate from potential quenchers and photo-oxidizers [39–42].

### Toxicity of SA-FSNPs

**Fig. 6:**
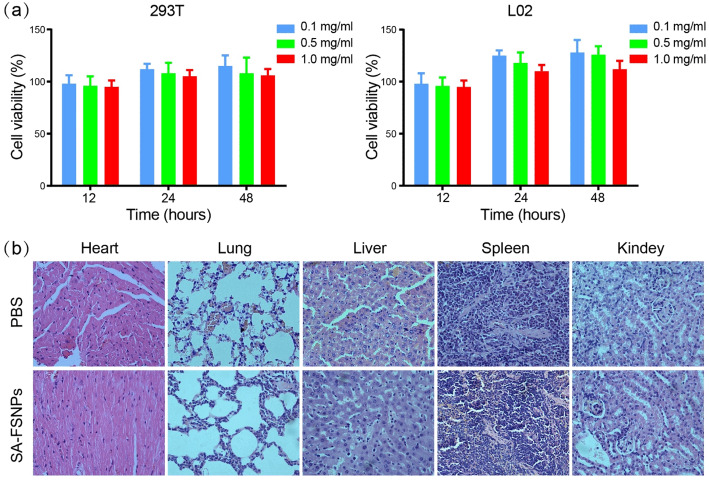
Toxicity of SA-FSNPs. **a** 293T and L02 cells in culture were incubated with various concentrations of SA-FSNPs, and cell viability was measured at 12, 24, and 48 h. **b** Nude mice were treated with PBS or SA-FSNPs, and sections from major organs were stained with hematoxylin-eosin and examined by light microscopy. Magnification, ×400

We assessed the cytotoxicity of SA-FSNPs on cultures of the normal cell lines 293T and L02. Viability of both cell lines was high according to the MTT assay after incubation with various SA-FSNP concentrations (Fig. 6a), suggesting that SA-FSNP showed minimal cytotoxicity. However, FSNPs have a short half-life in the circulatory system, and the entry of fluorescent dye molecules into the blood may increase the risk of systemic toxicity [43]. Therefore, it is necessary to evaluate the toxicity of SA-FSNPs in vivo. We further studied the in vivo toxicity of SA-FSNPs in nude mice. After intravenous injection of SA-FSNPs for 1 week, tissue sections of the main organs were stained with HE. As shown in Fig. 6b, there were no significant inflammation or necrosis observed on tissue sections. These results confirmed that SA-FSNPs were almost non-toxic to the main organs, showing the potential to be clinically useful as a diagnostic probe.

## Conclusions

We have developed an approach to detect hepatoma cells based on a biotin-labeled aptamer and streptavidin-modified FSNPs. The strong affinity and specificity of biotin-TLS11a for HepG2 tumor cells, coupled with the affinity and specificity of biotin for the streptavidin in SA-FSNPs, ensure highly specific and sensitive HepG2 detection. In addition, the fluorescence signal from SA-FSNPs is much stronger and more photostable than the signal from the FITC-labeled aptamer. SA-FSNPs do not show obvious toxic effects in vitro or in nude mice, based on the MTT assay or histology of major organs. This two-step labeling system may be adaptable to the detection of other cancers by changing the aptamer. In addition, this system may become a useful platform for targeted therapy if the nanoparticles can be loaded with anti-tumor drugs or microRNAs.

## Abbreviations

APTES: (3-aminopropyl) triethoxysilane
APTMS: 3-aminopropylmethyldimethoxysilane
Bio-TLS11a: Biotin-conjugated aptamer TLS11a
BSA: Bovine serum albumin
DAPI: 4′,6-diamidino-2-phenylindole
DMSO: Dimethyl sulfoxide
EDC: 1-ethyl-3-(3-dimethylaminopropyl) carbodiimide hydrochloride
FITC: Fluorescein isothiocyanate
FSNPs: Fluorescent silica nanoparticles
HE: Hematoxylin-eosin
MTT: 3-(4,5-dimethylthiazol-2-yl)-2,5-diphenyltetrazolium bromide
OD: Optical density
SA-FSNPs: Streptavidin-conjugated fluorescent silica nanoparticles
Sulfo-NHS: N-hydroxysulfosuccinimide sodium salt
TEM: Transmission electron microscopy
TEOS: Tetraethyl orthosilicate
